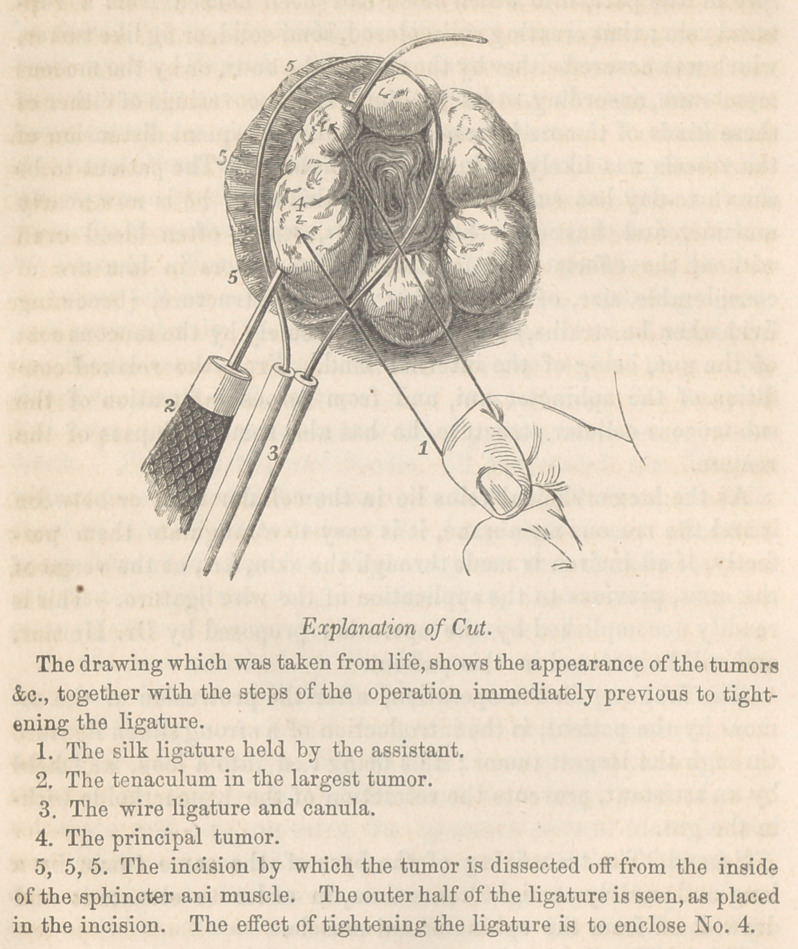# Summary of the Course on Demonstrative Surgery, in the University of Pennsylvania, from Jan. 25th to Feb. 22d

**Published:** 1851-03

**Authors:** 


### *Summary of the Course on Demonstrative Surgery, in the University of Pennsylvania, from Jan. 25th to Feb. 22d.*

(Reported for the Examiner.)

In continuing our report of the cases shown in the Demonstrative Surgical lectures of this institution, we shall be guided by the principle laid down in a previous paper, (Examiner for Feb., 1851,) and limit ourselves to such of the patients as present points either of novelty or general interest.

*Large Hydrocele of the Tunica Vaginalis Testis.*

A man upwards of 65 years of age, laboring under a large scrotal tumor of the right side, was brought before the class Jan. 25th, by Dr. Smith. As an apparently similar tumor had been shown by Dr. Horner on the previous Saturday, much stress was laid by the lecturer, on the means of diagnosis. This patient was believed to have a pure hydrocele, its existence having been rendered almost certain by an examination of the tumor with transmitted light. To effect this, the patient had been placed in a dark room, the tumor firmly grasped by one hand and rendered tense, whilst the other was placed breadth-wise on the top of the swelling, in order to cut off the rays of light from the eye of the observer. A lighted candle being now held by an assistant, on the opposite or sound side of the scrotum, and as near to the part as is possible, the contents had the appearance of a thin fluid, of a clear translucent character, presenting a light pink color, similar to that produced by looking at a light whilst the fingers are interposed between it and the eye. The testicle being a more solid body, could be distinctly seen like a dark mass at the posterior part of the tumor. When in such tumors light is not thus transmitted, (as was the case in the patient shown last week,) there is good reason to anticipate the existence either of hernia, hæmatocele, sarcocele or a thickened tunica vaginalis. The test, therefore, was regarded by Dr. Smith as a valuable one, and the more so, as long experience had not impaired its value.

On tapping the patient Dr. S. drew off upwards of three pints of clear, straw-colored serum; after which diluted tincture of iodine (iodine one part, water two parts,) was injected, and a few threads of silk passed through the cavity, in order to prevent the re-accumulation of the fluid before adhesion could occur. The man subsequently did well.

*Bad Hæmorrhoids—operated on by Dr. Horner's plan.*

On the removal of the last patient, reference was made by Dr. Smith, to a case of severe hæmorrhoids, accompanied by prolapsus of the rectum, on which he proposed operating. In explanation of the means to be employed in this case, the lecturer referred to the two well known methods of applying the ligature. 1st. That of the double waxed thread, introduced through the centre of the pile, cut into two pieces, and then one half tied on each side of the tumor ; and 2d. The destruction of the hæmorrhoids, by the double canula and wire ligature, as practised by Dr. Physick. In both instances strangulation was only indirectly accomplished, the circulation through the tumor being cut off, by constricting the vessels through the surrounding tissue. From the density of the part, perfect strangulation in this way was by no means easy, and the patient often suffered intensely, from the nerves of the skin, &c. being included within the ligature. For many hours after these operations, he was therefore left in a most miserable condition, suffering agony such as could only be appreciated by those who had witnessed it. Tetanic and other convulsions, were reported to have occasionally ensued, and, in more than one instance, the surgeon had been compelled to remove the ligature, in order to avert death. Petit, Kirby, and Brodie, all had stated the dangers which sometimes ensued on these operations, and Petit reported the death of one of his patients within forty eight hours after this application of the ligature. Most surgeons were therefore so well acquainted with the danger referred to, that caution in the strangulation of hæmorrhoids was always urged.

In the plan proposed by Dr. Horner, and published in the American Journal of Medical Sciences for October, 1842, most of the suffering, and all such dangers were entirely removed, and as the Dr. had now operated in this manner more than thirty times, as the lecturer had assisted him in a large proportion of the cases, and also repeated the operation several times in his own practice, he could confidently recommend it. Out of all the cases that he had known, but one, (a cavalry officer) had required its repetition, and in most instances the tumors and ligature were removed, in five hours after the operation, without causing any subsequent bad symptoms.

In referring to the structure of hæmorrhoids, as well as to the objects of the operation, Dr. Smith expressed the belief, that in most instances the production of the tumors was due, not only to a varicose condition of the plexus hæmorrhoidalis, &c., but also to the extension and protrusion of these vessels, covered by the mucous membrane of the gut, in the expulsive efforts consequent on defecation. In other instances, and especially in external piles, the tumors were, as he thought, simply the cellular structure of the part, into which blood had been effused from a ruptured vein; thus creating a discolored, semi-solid, or fig like tumor, which was covered either by the skin of the anus, or by the mucous membrane, according to its position. If the coverings of either of these kinds of tumors became ulcerated, subsequent distension of the vessels was likely to induce hæmorrhage. The patient to be shown to-day has suffered severely in this way; he is now nearly anæmic, and has open hæmorrhoids, which often bleed even without the efforts of defecation. The tumors in him are of considerable size, of a somewhat vascular structure, (becoming livid when he strains,) and are covered solely by the mucous coat of the gut, being of the internal kind. From the relaxed condition of the sphincter ani, and from serous infiltration of the sub-mucous cellular structure, he has also some prolapsus of the rectum.

As the hæmorrhoidal veins lie in the cellular coat, or between it and the mucous membrane, it is easy to strangulate them perfectly, if an incison is made through the skin, &c., at the verge of the anus, previous to the application of the wire ligature. This is readily accomplished by the operation proposed by Dr. Horner, and will be pursued in this patient.

The first step in the operation, after the protrusion of the tumors by the patient, is the introduction of a strong silken ligature through the largest tumor; this being tied into a loop, and held by an assistant, prevents the retraction of the hæmorrhoids within the gut.

Second. The transfixing of the base of the same tumor by a long and nearly straight tenaculum, in order to elevate it and draw it off from the sphincter ani muscle.

Third. The division by a scalpel, of the skin at the base of the tumor, or at the line where the mucous membrane merges into the skin of the anus, and the free dissection of the pile from the inner circle of the fibres of the sphincter ani.

Fourth. The application of a wire ligature and canula, in such a manner that one half of the loop is passed to the bottom of the incision made on the pelvic side of the pile, whilst the other is pressed up the gut on the inside, until it reaches above the upper portion of the base of the tumor.

On drawing the ligature very firmly, the veins will be constricted on the outer side, through the cellular coat, or between it and the muscle; and on the inner side, through the mucous membrane. The tumor therefore, very soon becomes livid, and the strangulation is so perfect, that in five hours both it and the ligature may be removed by excision, without hæmorrhage. The thread left in the tumor, renders its excision easy; after which the loop of the wire ligature can be readily slipped off the stem-like portion.

The steps taken immediately after the operation, are the removal of the tenaculum; the introduction of a piece of spread cerate into the incision, so as to separate the outside of the tumor from the incised parts, to which it would otherwise be disposed to unite; and the administration of an anodyne enema (laudanum ʒj for an adult). For the first 24 or 48 hours, it is also often necessary to employ the catheter. The wound usually heals without further dressing. If the tumors are small, two or three of them may be included in the same operation.

In no instance within the knowledge of the lecturer had there been subsequent hæmorrhage, phlebitis or other bad symptom, whilst the contraction of the cicatrix usually produced sufficient constriction about the anus, to relieve the tendency to relaxation of the part, which is so often co-existent with the disease, and doubtless tends to its reproduction.

The patient being then etherised, was operated on by Dr. Smith, in the manner stated, and in twelve days was able to walk several squares to his home. Although in this, as in other cases, the hæmorrhoids are found all around the anus, the performance of the operation on one side, generally effects their cure.

### *Psoriasis Inveterata.*

In connection with the treatment of a patient laboring under this complaint, Dr. Smith stated that for some years he had seen much benefit in such cases, from the use of the tinctura cantharidis, as proposed by Biett. He gave it in doses varying from ten to fifty drops three times a day, and at the same time employed Hydrargyrum Prot. Iodidum, as an ointment, in the proportion of grs. xii. to the ounce of lard.

### *Ulcerated Cancer of the Mamma.*

On the 1st of February Dr. Horner brought forward a lady laboring under ulcerated or open cancer of the right mammary gland, of two years duration. This patient had been under the care of Homœopathic practitioners for many months, but finding that she rapidly grew worse, had recently sought better advice.

The breast now was flattened and firmly adherent to the pectoral muscle; presented an ulcer of considerable size, with thick everted edges; sanious pus; wrinkled and contracted skin, enlargement of the axillary glands, and all the well known signs of advanced cancer. An operation being therefore unadvisable, local and constitutional treatment were resorted to, and she was directed to dress it constantly with the powdered carbonate of iron and dry lint, and to take Vallet's mass. grs. ij. twice or thrice daily. This treatment was thought to offer the best means of arresting the progress of the disease, and had formerly been employed to a considerable extent by Cline, Justamond, and others.

Sufficient time has not yet elapsed to test the result in this case, but in some others in the city, the chalybeate course has proved decidedly beneficial, especially in the early stages of the complaint.

Cases of aneurism of the aorta just above the heart; of fatty tumor of the shoulder ; caries and removal of the 3rd metatarsal bone, &c., were presented Feb. 8th, but require no further notice.

*Section of the Frontal Nerve for Neuralgia.*

Feb. 15th. Dr. Horner brought forward a female who had labored for five or six months under severe neuralgia of the forehead and eye, believed to arise from disease of the branches of the frontal nerve. This patient was about 35 years of age, and had been treated by all the usual remedies, as blisters, quinine, iron, &c., without benefit. It had therefore been decided to divide the main trunk of the nerve, by a sub-cutaneous incision, at its exit from the supra-orbitar foramen. Accordingly, the Doctor introduced a narrow sharp-pointed knife, flat-wise beneath the integuments, a little to the external side of the supra-orbitar foramen, and passing it close to the cranium, turned its edge forwards so as to cut from behind, to near the surface of the skin ; then turning the blade again upon its side, withdrew it at the point of entrance, the puncture being immediately closed. Directly after the incision, the patient declared herself free from pain, and that sensation in the part was destroyed. The necessary division of the supra-orbital artery, caused slight hæmorrhage, but it readily yielded to the pressure of a bandage.

### *Club Foot.*

Dr. Horner also presented a child with double club foot, (varus and pes equinus,) which had been treated by Dr. Smith, by mechanical means, and subcutaneous division of the tendo-Achillis, three years since. The object in showing this case was to call attention to the necessity of continuing the use of the shoes, until the tarsal bones were fully ossified—probably about 16 or 18 years of age. This child's foot appeared perfectly well when the shoes were on, and it ran about without difficulty, being very active, yet on the removal of the apparatus, although the soles were flat, the child was decidedly pigeon-toed.

### *Refracture of the Leg—to relieve a Deformity.*

On the removal of the other patients, Dr. Horner presented a young man from the country, who, twelve weeks since had received a fracture in both bones of his leg, in jumping from a railroad-car, whilst it was in motion. For some time after the accident the leg appeared straight, but subsequently was found to be very much out of line, projecting anteriorly, the limb being shortened about an inch. Fortunately for the patient, although there was a large amount of provisional callus, the union was not perfect, the fracture yielding on the application of force. It was therefore determined to fracture the callus and endeavor to give the man a more useful limb. Accordingly the patient was partially etherised, and a thickly padded splint applied to the back of the limb, from the seat of fracture up to the pelvis. Two splints also well padded, were then fastened to the sides of the leg so as to reach from a short distance below the fracture nearly three feet beyond the heel, and the limb being brought to the edge of the table the foot was forcibly bent backwards, and the fracture reproduced, by carefully acting on the side splints as simple levers. The limb being thus straightened, was then confined in splints, and the patient has now every prospect of recovery with a good leg. No fever or other bad symptom supervened on the operation.

### *Rare dislocation of the Femur.*

On the 22nd of February, Dr. Horner brought forward a man laboring under a dislocation of the head of the os femoris *in front*

*of the spinous process of the ischium*, (or just above the tuber ischii, between it and the spine,) of six weeks standing. After applying two sets of pullies, one to make extension, and the other vertically, so as to lift the head of the femur over the prominent edge of the acetabulum, the patient was most happily relieved, and the bone replaced in its articulating cavity. The symptoms, &c., of this case offered so much that seems worthy of record that we shall reserve a full account of them for a future paper. A case of encysted tumor of the ham, resembling popliteal aneurism; one of paralysis of the flexor muscles of the fore-arm, from the patient having slept with the arm under his head; one of large goitre; one of hygroma of the lower eye lid; and one of fungus of the testicle, were also presented, but were reserved for future treatment from the want of time on this occasion.

## Figures and Tables

**Figure f1:**